# Circulating Tumor DNA Detection in Early-Stage Non-Small Cell Lung Cancer Patients by Targeted Sequencing

**DOI:** 10.1038/srep31985

**Published:** 2016-08-24

**Authors:** Ke-Zhong Chen, Feng Lou, Fan Yang, Jing-Bo Zhang, Hua Ye, Wei Chen, Tian Guan, Ming-Yu Zhao, Xue-Xia Su, Rong Shi, Lindsey Jones, Xue F. Huang, Si-Yi Chen, Jun Wang

**Affiliations:** 1 Department of Thoracic Surgery, Peking University People’s Hospital, Beijing, China; 2 Department of Bioinformatics, San Valley Biotechnology Incorporated, Beijing, China; 3 Departments of Molecular Microbiology & Immunology and Norris Comprehensive Cancer Center, Keck School of Medicine, University of Southern California, Los Angeles, CA, USA

## Abstract

Circulating tumor DNA (ctDNA) isolated from peripheral blood has recently been shown to be an alternative source to detect gene mutations in primary tumors; however, most previous studies have focused on advanced stage cancers, and few have evaluated ctDNA detection in early-stage lung cancer. In the present study, blood and tumor samples were collected prospectively from 58 early-stage non-small lung cancer (NSCLC) patients (stages IA, IB, and IIA) and a targeted sequencing approach was used to detect somatic driver mutations in matched tumor DNA (tDNA) and plasma ctDNA. We identified frequent driver mutations in plasma ctDNA and tDNA in *EGFR*, *KRAS*, *PIK3CA*, and *TP53*, and less frequent mutations in other genes, with an overall study concordance of 50.4% and sensitivity and specificity of 53.8% and 47.3%, respectively. Cell-free (cfDNA) concentrations were found to be significantly associated with some clinical features, including tumor stage and subtype. Importantly, the presence of cfDNA had a higher positive predictive value than that of currently used protein tumor biomarkers. This study demonstrates the feasibility of identifying plasma ctDNA mutations in the earliest stage lung cancer patients via targeted sequencing, demonstrating a potential utility of targeted sequencing of ctDNA in the clinical management of NSCLC.

Lung cancer is the most common cancer in the world and leading cause of cancer-related deaths accounting for nearly 1.6 million deaths in 2012^1^,^2^. The most effective management and the highest chance of survival is achieved when the cancer is identified and treated early; however, only 30% of lung cancers are diagnosed at an early stage^3^. Patients with stage IA non-small cell lung cancer (NSCLC) have up to a 73% 5-year survival rate, whereas 5-year survival falls to 24% for those with stage IIIA NSCLC^4^.

While early detection is critical in the fight against cancer, this may be a challenge in some lung cancer patients. For example, the physical tumor location in the lungs may make biopsies difficult if not impossible to obtain. Imaging diagnostics have difficulties distinguishing between benign and malignant lesions, and these difficulties become more pronounced for small lesions^5^. Expression of tumor markers such as carcinoembryonic antigen (CEA), carbohydrate antigen 19-9 (CA19-9), carbohydrate antigen (CA125), cytokeratin 19 fragment (CYFRA21-1), and neuron-specific enolase (NSE) in serum have also been used to aid in cancer diagnoses, but these markers are not specific to lung cancer and may be associated with other pathologies^6^,^7^. Thus, minimally invasive diagnostic techniques are continually being investigated.

Recently, studies have shown that DNA released from lysed or apoptotic tumor cells circulate freely in blood plasma, referred to here as circulating tumor DNA (ctDNA), can be isolated to aid in cancer treatment^8^,^9^,^10^,^11^. Even small tumors containing as few as 50 million cells release sufficient DNA to be detected in the blood, whereas tumors of this size fall well below the detection limit of standard radiological techniques^12^. Since ctDNA comprises a small, variable fraction of total DNA circulating in the blood known as cell-free DNA (cfDNA), and mutant DNA molecules account for 0.02% to 0.1% of all the DNA assayed^13^, sensitive methods are necessary to identify mutations in this small fraction. Recent studies have used BEAMing (beads, emulsion, amplification and magnetics), peptide nucleic acid (PNA)-mediated polymerase chain reaction clamping method, and commercial kits such as the cobas *EGFR* mutation test and Scorpion ARMS-based *EGFR* mutation detection kit to successfully detect genetic changes in ctDNA from lung cancer patients^14^,^15^,^16^,^17^.

Despite recent advances, most previous studies identifying mutations in ctDNA from lung cancer patients focus on advanced-stage cancers and analyze a limited number of genes^18^,^19^, particularly *EGFR* and *KRAS* as these are frequently mutated in lung cancers and have implications in targeted therapies^14^,^20^. The next-generation sequencing Ion Personal Genome Machine (PGM) is capable of screening for mutations in multiple genes simultaneously^21^, which may be useful in the clinical characterization of plasma ctDNA from lung cancer patients. Therefore, the aim of this prospective study was to evaluate the feasibility of utilizing a targeted DNA sequencing approach with the Ion PGM and AmpliSeq Cancer Panel to detect mutations in 50 cancer-related genes in matched plasma ctDNA and tumor DNA (tDNA) samples from 58 early-stage NSCLC patients.

## Results

### Sequencing coverage and internal validation

For all matched tDNA and plasma ctDNA samples analyzed, the sequence length distribution nearly fit the normal distribution. The vast majority of read lengths were between 60 to 160 bp; therefore, sequences within this range were selected for further analysis. The GC content across all bases was found to be roughly 50%; however, because the GC content between 1 and 20 bp fluctuated drastically, this region of reads was removed during quality control. The quality scores across all bases signify the sequencing accuracy. For all plasma ctDNA samples, the depth of most amplicons was over 10,000x (Supplementary Fig. S1). In addition to the tDNA and plasma ctDNA from NSCLC patients, the serum from four disease-free individuals was screened using our 50-gene cancer panel for internal validation. We determined that all mutations identified in these four samples were germline mutations (Supplementary Table S1). Furthermore, we constructed positive and negative accuracy plasmids with either specified mutations or as wild type and determined the accuracy of our sequencing method to be 100%.

### Concordance in tDNA and plasma ctDNA sample pairs

A total of 58 tDNA and ctDNA sample pairs were analyzed (clinical patient characteristics can be found in Table 1). We found that 45 (77.6%) contained mutations in one or more of the 50 genes screened in either tDNA or plasma ctDNA, or in both sample type, and of these the average number of mutations per sample pair was 2.24. A total of 135 mutations were identified, including 59 in tDNA and 76 in plasma ctDNA, and 34 concordant mutations in both tDNA and plasma ctDNA. Twenty-three sample pairs (39.7%) had concordant mutations in both tDNA and plasma ctDNA (Supplementary Table S2), and the average variant frequency in these plasma ctDNA samples was 1.29%. Thirteen sample pairs (22.4%) had no mutations in any of these 50 genes in either sample type. Ten sample pairs had tDNA mutations only (Supplementary Table S3), and eight sample pairs had plasma ctDNA mutations only (Supplementary Table S4). Four sample pairs had mutations in both tDNA and plasma ctDNA but the mutations were different. Eleven sample pairs with concordant mutations contained additional discordant mutations in either sample type (Supplementary Tables S3 and S4). For the 19 plasma ctDNA samples with mutations not found in tDNA the average variant frequency was 2.72%.

**Figure 1:**
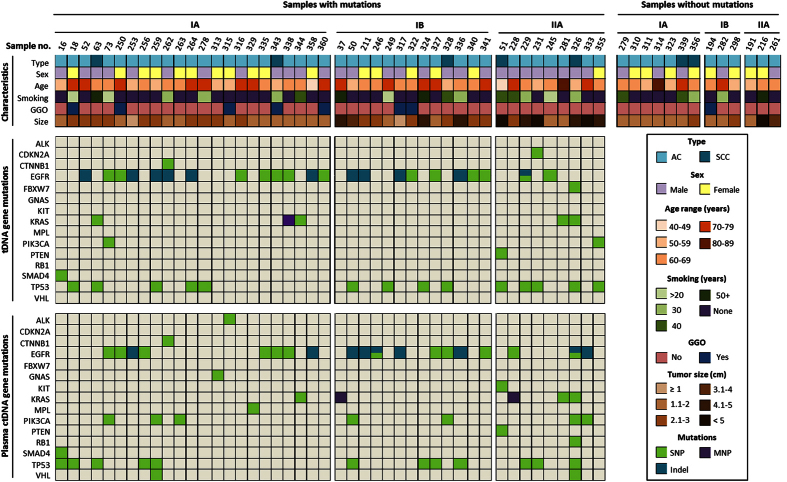
Summary of patient characteristics and gene mutations in matched tDNA and plasma ctDNA sample pairs. Samples are classified by the following methods: 1) AJCC stage (IA, IB, IIA); 2) pathologic diagnosis; 3) sex; 4) age range; 5) smoking history; 6) GGO; and tumor size (top). Mutation type (SNP, MNP, or Indel) per sample in tDNA (middle) is compared to those found in plasma ctDNA (bottom). Samples without mutations in the 50 genes screened are shown on the top right.

Collectively 27 sample pairs were positive for both tDNA and ctDNA mutations. The overall concordance rate between tDNA and matched plasma ctDNA mutations was 50.4%, with a sensitivity of 53.8% (95% CI: 35.3%–71.5%), specificity of 47.3% (95% CI: 28.6%–66.8%), and a plasma positive predictive value (PPV) of 53.2% (95% CI: 34.8%–70.8%). In addition to tDNA and plasma ctDNA, white blood cell (WBC) DNA from each patient was also analyzed and no somatic mutations were identified in any of the samples, indicating both the low background level in the sequencing reads and reliability of the variant calling. Fig. 1 illustrates the specific mutation profile of each of the 45 sample pairs with mutations along with clinical patient features, and also the clinical features of the 13 patients with no identified mutations.

### Mutations in plasma ctDNA and tDNA samples

**Figure 2:**
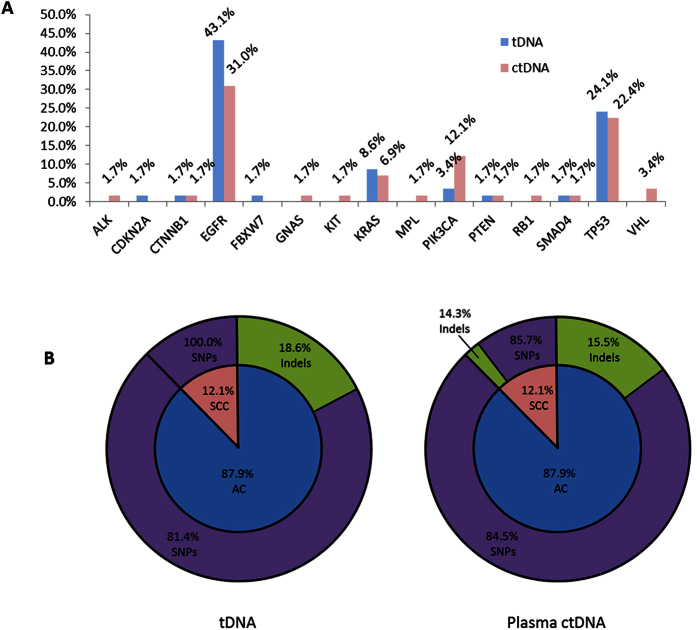
Comparison of the distribution of driver mutations identified in 58 matched tDNA and plasma ctDNA samples. (**A**) Rates of gene mutations in tDNA vs. plasma ctDNA from matched sample pairs; (**B**) Pie chart shows the distribution of mutation types identified in matched sample pairs of tDNA (left) and plasma ctDNA (right). Inner circle shows distribution of cancer type (AC and SCC), outer ring shows frequency of mutation type (SNP vs. Indel) per respective cancer type.

Including concordant and discordant mutations between sample pairs, mutations were identified in tDNA and/or plasma ctDNA samples in 15 of the 50 genes screened in the cancer panel (Fig. 2A). Collectively, mutations in *EGFR* were the most common and were found in 39.7% of tDNA samples and 32.8% of plasma ctDNA samples. Other genes frequently mutated were *TP53*, *PIK3CA*, and *KRAS*. Interestingly, *TP53* and *KRAS* mutations were found in roughly equal proportions of tDNA and plasma ctDNA samples, whereas *PIK3CA* mutations were nearly four-fold higher in plasma ctDNA compared to tDNA.

In all 58 tDNA and plasma ctDNA sample pairs, the majority of all mutations identified in both tDNA and plasma ctDNA were single nucleotide polymorphisms (SNPs) (78.9% and 81.7%, respectively), insertions and deletions (Indels) accounted for 19.3% of tDNA mutations and 15.5% plasma ctDNA mutations, and 1.8% of tDNA and 2.8% of plasma ctDNA mutations were multi-nucleotide polymorphisms (MNPs) (Fig. 2B). Indels were only found in *EGFR* and MNPs were found only in *KRAS*. Additionally, Indels in tDNA were only found in AC samples, whereas 14.3% of SCC samples contained Indels.

### Clinical features and cfDNA concentration

We investigated the correlation between a series of clinicopathological variables including age, sex, smoking history, tumor histology, stage, differentiation level, ground glass opacity (GGO), vascular invasion, and visceral pleural involvement (VPI) with cfDNA concentration in NSCLC patients (Table 2). Patients with GGO-dominant tumors (GGO proportion > 50%) had plasma cfDNA concentrations more than ten times lower than those with solid-dominant tumors (average 0.66 ng/ml vs. 7.53 ng/ml, respectively; *p* = 0.050). Additionally, stage II patients had significantly higher plasma cfDNA concentrations than stage I patients (average 14.28 ng/ml vs. 4.57 ng/ml, respectively; *p* = 0.050). There were no associations found between cfDNA concentrations and any of the other clinicopathological variables analyzed.

### Detection of cfDNA versus serum tumor markers

Plasma samples were analyzed for the presence of the tumor biomarkers CA125, CA19-9, CEA, CYFRA21-1, and NSE. Analysis revealed that no plasma samples were positive for serum CA125, two were positive for CA19-9, eight were positive for CEA, 16 were positive for CYFRA21-1, and four were positive for NSE (Supplementary Table S5). Collectively, 25 (43.1%) plasma samples were positive for these five tumor markers. In contrast, 52 (89.7%) plasma samples were positive by cfDNA detection. Compared to these five serum tumor biomarkers, cfDNA has a higher PPV for early stage NSCLC.

## Discussion

The present prospective study examined the possibility of using a targeted sequencing approach to identify plasma ctDNA mutations in early-stage NSCLC patients with the Ion PGM and AmpliSeq Cancer Panel. To our knowledge, this study is the first prospective study to assess the possibility of detecting ctDNA mutations by targeted sequencing in plasma samples from patients with early-stage NSCLC. Overall, 52 (89.7%) of patients were found to have quantifiable cfDNA, suggesting that identifying the presence of cfDNA may be a good indicator of cancer. Plasma ctDNA mutations were identified in 35 (60.3%) of the 58 patient samples, and identified plasma ctDNA and tDNA mutations had 50.4% concordance, sensitivity and specificity of 53.8% and 47.3%, respectively, and a PPV of 53.2%. Collectively these results suggest that targeted sequencing of ctDNA may have potential value in the clinical management of NSCLC. However, because only four healthy individuals were used as negative controls in this study for experimental validation, further investigation with larger populations of diseased and disease-free individuals is warranted to establish clinical sensitivity and specificity with confidence.

More mutations were found in plasma ctDNA samples than in tDNA (76 versus 58, respectively) despite the roughly equal number of discordant mutations per sample type (20 sample pairs with unmatched tDNA mutations versus 19 sample pairs with unmatched plasma ctDNA mutations). One SCC sample from an individual who smoked for 30 years had 14 plasma ctDNA mutations not found in tDNA, and this sample alone lowered the overall study concordance by more than 4%. It has been reported that necrotic WBCs release genomic DNA into the blood, and this may dilute the ctDNA fraction in the plasma making detection of ctDNA more challenging^13^. This may have been a contributing factor in our study in the discordant sample pairs with mutations identified in tDNA but not in plasma ctDNA. Genomic DNA may also cause technical challenges as others have shown that delayed processing of the blood samples can reduce the mutation detection rate, possibly due to WBC necrosis^22^. To control for this, all blood samples used in our study were processed immediately to eliminate this variable. A consideration for future studies would be the use of two separate blood samples obtained from the same individual at once and purified in parallel to provide additional confirmation of identified plasma ctDNA mutations. Blood samples obtained at a later time would not necessarily be used to confirm plasma ctDNA mutations but to track changes in detectable mutations. As the goal of this study was not to monitor the ctDNA mutation status over time, we only obtained blood samples from the participants at the time of surgery. Additional studies analyzing the mutations of early-stage cancer patients before and after surgery would be of great value to track the change in mutation status after treatment and further support the use of this targeted sequencing approach in the clinical management of cancer.

In addition to genomic DNA or technical isssues, the discrepancy of mutations found in plasma ctDNA but not tDNA may be attributed to the heterogeneous nature of lung tumors and reflect the limitations that are intrinsic to biopsies or tissue sections that only sample a small portion of the tumor which may not reflect the characteristics of the entire tumor^23^,^24^, and tissue sections were used for sequencing analysis in our study. Unfortunately limited tumor tissues were available to us for use in this study, therefore dissecting and sequencing different parts of the tumor was not possible. This would be a useful strategy in future studies to determine if mutations found in plasma ctDNA only were due to sequencing only part of the tumor; however, the small size of many early-stage tumors may be a limiting factor in this approach.

Samples with only concordant mutations between sample type had an average size of 1.94 cm, samples with tDNA mutations only were an average of 2.48 cm, and samples with ctDNA mutations only were an average of 2.84 cm. Interestingly two samples with concordant, ctDNA only, and tDNA only mutations were an average of 5.75 cm, both SCC, and from smokers of 30+ years. Of the 36 stage I samples with mutations, 52.8% had concordant mutations and 69.4% had discordant mutations; of 9 stage IIA samples with mutations, 55.6% had concordant mutations but 88.9% had discordant mutations, 55.6% of which also had concordant mutations. Taken together, these results suggest that concordance may be influenced by tumor size, but also that tissue sections from larger tumors may be less representative of the whole tumor causing false negative tDNA results and unmatched ctDNA mutations. Smoking history may also influence mutation concordance, for we found that 16.7% of those with only concordant mutations, 43.5% of those with concordant and discordant mutations, and 31.8% of those with discordant only mutations had a history of smoking.

Previous reports show that ctDNA mutations from primary tumors typically have a variant allele frequency of only 0.1–10.0%, even in samples from patients with advanced cancers^25^,^26^. Nearly all plasma ctDNA samples in our study had variant frequencies within this range, and interestingly the variant frequency of mutations found in plasma ctDNA only was twice as high as that of mutations found in both sample types (2.72% versus 1.29%, respectively). Two plasma ctDNA samples had high mutant variant frequencies of 12.3% and 15.8%, where the former was not found in corresponding tDNA. That this mutation with such a high variant frequency was not identified in the tumor tissue may again suggest that this clone was not captured in the tissue section used for sequencing analysis.

Mutations were not detected in plasma ctDNA or tDNA samples from 13 patients, but this may reflect a limitation of the cancer panel of 50 defined genes screened in this study. The stages of patients without mutations were in roughly equal proportions to those with mutations (53.8% vs. 51.1% stage IA, respectively; 23.1% vs. 28.9% stage IB, respectively; and 23.1% vs. 20.0% stage IIA, respectively), indicating that the lack of identified mutations was not related to the cancer stage in our study. Also in roughly equal proportions were the smokers vs. non-smokers in those with or without mutations (37.8% with mutations smoked vs. 38.5% without mutations smoked). Only one (7.7%) of the patients without mutations had a GGO-dominant tumor whereas seven (15.5%) of those with mutations had GGO-dominant tumors. Considering these results, it is possible that these 13 patients have rare mutations that were not covered in our 50-gene panel.

Measuring tumor biomarkers in patient serum has been shown to be a convenient way to monitor disease progression over time, but as these markers lack specificity to a single cancer type, they are rarely used for diagnostic purposes. Furthermore, as the concentration of these biomarkers in serum is related to tumor stage and size, detection can be a challenge in early-stage cancer patients^27^. In our study cfDNA was found in a higher proportion of samples with a higher PPV for early-stage cancer compared to the analyzed tumor biomarkers, suggesting that detecting cfDNA by targeted sequencing is a more sensitive method than evaluating certain serum tumor biomarkers by ELISA.

Greater than 30% of patients with stage I NSCLC die from relapse or metastasis after complete surgical resection^28^. Why this subset of early-stage NSCLC patients relapse while others do not is still unclear. Sozzi *et al*. observed a correlation between baseline cfDNA and aggressive disease in patients with stage IB—IV NSCLC, suggesting that pre-surgery cfDNA levels could be indicative of a patient’s prognosis^29^. Sirera *et al*. found that high pre-treatment cfDNA levels acted as independent prognostic markers for short survival rates^30^, whereas another study did not find any correlation between cfDNA levels and survival^31^. Cancer-specific ctDNA mutations were used as biomarkers in our study and our analysis of the correlation between cfDNA levels and clinical features showed that the amount of cfDNA is associated with cancer stage.

Studies have reported excellent survival after resection of pure GGO lesions, which is markedly better than that reported for solid lung cancer. These sub-solid lesions have indolent behavior and are mostly pre-invasive lesions, including adenocarcinoma *in situ* (AIS) and minimally invasive adenocarcinoma (MIA), whereas more advanced ACs may include a larger solid component within the nodules^32^. We compared patients with GGO-dominant lesions to patients with solid-dominant lesions, and the results showed that there were significant differences in cfDNA concentrations between the two groups. The results of our study imply that the level of cfDNA reflects the behavior of NSCLC, where indolent GGO-dominant tumors which are mostly either pre-invasive or lepidic-predominant ACs release relatively less cfDNA, while more aggressive ACs, such as solid-predominant AC, release considerably more.

In conclusion, this study demonstrates that targeted sequencing with the Ion PGM and AmpliSeq cancer panel with broad coverage of known driver mutations can detect ctDNA mutations in the plasma of early-stage NSCLC patients with concordance to mutations found in primary tumor tissue and greater sensitivity than detecting the expression of various tumor markers. We found that the amount of cfDNA is associated with the cancer stage and tumor aggressiveness. Thus this sequencing approach could easily be implemented and standardized for clinical use in the diagnostic and prognostic assessment of patients with early-stage NSCLC.

## Methods

### Study Design and Patients

The protocols of this study (ClinicalTrials.gov identifier NCT02645318) were approved and carried out in accordance with the approved guidelines by the Institutional Review Board of Peking University People’s Hospital. Written informed consent was obtained from all subjects for the use of the blood and resected tumor tissue for research purposes prior to surgery. All samples and medical data used in this study have been irreversibly anonymized.

During May 2015 to July 2015, consecutive cases of patients with suspected NSCLC who underwent curative-intent resection were eligible to enter the study. Patients who received any treatment prior to resection or had a history of malignancy were excluded. All blood samples were obtained just prior to surgery and were immediately processed to isolate plasma. Tumor samples were collected during the surgery when the frozen section confirmed malignancy. As a result, 95 patients were enrolled and the paired samples were processed to DNA extraction and sequencing before the paraffin pathology and stage were known. Five patients were excluded when peripheral blood samples were not collected from two patients and ctDNA extraction failed in three samples (two serum and one tissue sample). Additionally, the formalin-fixed, paraffin embedded (FFPE) tumor section revealed three cases of small cell carcinoma, one carcinoid, two unclassified carcinomas, and 26 pathological stage IIB cancers, all of which were then excluded from the analysis. In total, 58 patients diagnosed with stage IA, IB, or IIA adenocarcinoma or squamous carcinoma were included. In addition to diseased patients, the serum from four healthy, cancer-free patients was screened for mutations for experimental validation.

### Tumor Tissue and Blood Sample DNA Preparation

Fresh tumor tissue, peripheral blood cells, and plasma samples were collected from each patient. The E.Z.N.A. Tissue DNA kit (Omega Bio-Tek, Norcross, GA) was used to extract DNA from fresh tissue. Blood samples in EDTA tubes were centrifuged for 10 min at 1,600 g and the peripheral blood lymphocyte (PBL) debris was stored at −20 °C until use. The supernatants from these samples were further centrifuged at 16,000 g for 10 min, and plasma was collected and stored at −80 °C until needed. Following the manufacturers’ respective instructions, the E.Z.N.A. Blood DNA kit (Omega Bio-Tek) was used to extract DNA from PBLs and the QIAamp Circulating Nucleic Acid kit (QIAGEN) was used to extract cfDNA from 1 ml of plasma which was collected in 40 μl of eluent. The Qubit 2.0 Fluorometer and Qubit dsDNA HS Assay kit (Life Technologies, Carlsbad, CA) were used to quantify DNA following the recommended protocol.

### Ion PGM Library Preparation and Sequencing

Ion PGM library preparation, sequencing and variant calling was performed as described in our previous publications^33^,^34^,^35^. For each sample type, the Ion AmpliSeq Library Kit 2.0 (Life Technologies) was used to generate an Ion Torrent adapter-ligated library following the manufacturer’s protocol. In brief, 4~15 ng of pooled cfDNA amplicons were end-repaired, then DNA ligase was used to ligate the Ion Torrent adapters P1 and A. Adapter-ligated products were purified with AMPure beads (Beckman Coulter, Brea, CA), nick-translated, and PCR-amplified for a total of 5 cycles. The resulting library was purified with AMPure beads, and the concentration and size of the library was determined with an Agilent 2100 Bioanalyzer (Agilent Technologies) and Agilent Bioanalyzer DNA High-Sensitivity LabChip (Agilent Technologies).

For sample emulsion PCR and emulsion breaking, the Ion OneTouch system (Life Technologies) with the Ion PI Template OT2 200 Kit v3 (Life Technologies) were used following the manufacturer’s instructions. After recovering Ion Sphere Particles (ISPs), Dynabeads MyOne Streptavidin C1 beads (Life Technologies) were used to enrich template-positive ISPs on the Ion One Touch ES (enrichment system) (Life Technologies). The Qubit 2.0 Fluorometer was used to confirm ISP enrichment. Barcoded samples at a concentration of ~100 pM were sequenced on the Ion Proton using Ion PI v2 Chips (Life Technologies) for 100 cycles. Finally, sequencing reactions were performed using the Ion PI Sequencing 200 Kit v3 (Life Technologies) following the recommended protocol.

### Variant Calling

To determine the accuracy and minimum variant frequency threshold, the following method was used: a plasmid with 100% *KRAS* p.G12C, *PIK3CA* p.E545K, *PIK3CA* p.H1047R, *BRAF* p.V600E, *EGFR* p.L858R, and *EGFR* p.E746_A750delELREA mutations was used as a positive control, and the negative control was a wild type plasmid at these positions. HindIII enzyme sites were inserted into both plasmids allowing the plasmids to be divided into 180 bp DNA segments. These two plasmids were mixed in 0.0%, 0.1%, 0.5%, and 1.0% proportions of mutation reference standards. Each proportion of reference standard was sequenced 12 times using the 50-gene cancer panel with 10,000x sequencing depth. The results indicated that the accuracy was 100% and the detection limit of our sequencing method is 0.1%.

The Ion PGM platform-specific pipeline software Torrent Suite was used to process initial data from the PGM runs to generate sequence reads, trim adapter sequences, and filter and remove poor signal-profile reads as described in our previous publications^33^,^34^,^35^. The Torrent Suite Software v3.0 with a plug-in “variant caller v3.0” program was used to generate initial variant calling from the Ion AmpliSeq sequencing data. To remove erroneous base calling and generate final variant calling, three filtering steps were used. First, the following were defined for tissue samples: the average total coverage depth as >1000; each variant coverage as >20; a variant frequency of each sample >5%; and *p* value < 0.01, and the following were defined for plasma samples: the average total coverage depth as >10000; each variant coverage as >10; a variant frequency of each sample >0.1%; and *p* < 0.01. The second filter eliminated possible DNA strand-specific errors after visual examination of called mutations using Integrative Genomics Viewer (IGV) software (http//www.broadinstitute.org/igv) or Samtools software (http://samtools.sourceforge.net). The final filtering step set variants within the 739 mutational hotspots, as per the manufacturer’s instructions.

### Tumor Biomarker Analysis

In addition to mutation analysis with the Ion PGM and AmpliSeq cancer panel, plasma samples were analyzed for the tumor markers CA125, CA19-9, CEA, CYFRA21-1, and NSE using an electrochemiluminescence immunoassay (Cobas E601; Roche, Indianapolis, IN, USA), according to the manufacturer’s instructions. The following cut- off values were used to define the samples as negative for the respective tumor markers: CA125 < 35 U/ml, CA199 < 39 U/ml, CEA < 4.7 ng/ml, CYFRA21-1 < 3.3 ng/ml, NSE < 16.3 ng/ml. All measurements were performed in duplicate.

### Statistical Analysis

For statistical analysis, tumor DNA was used as the reference when compared to plasma ctDNA. True positives (TP) were defined as matched tDNA and plasma ctDNA samples with the same identified mutations, and true negatives (TN) were defined as matched sample pairs without mutations (wild type) in the 50 genes screened. False positives (FP) were defined as mutations identified in plasma ctDNA but not tDNA, whereas mutations found in tDNA but not plasma ctDNA were considered to be false negatives (FN). Several samples contained both concordant and discordant mutations, and some had multiple of these mutations. To maintain the correct total number of cases (n = 58) for statistical purposes, the samples with both concordant and discordant mutations, or with mutations in both sample type that were different, were assigned values based on the following formula: 1/the total number of mutations per sample pair = n × the number of mutations per statistical group (TP, FP, FN). For example, sample 259 had three ctDNA only mutations and two tDNA only mutations so it was calculated as follows: 3 + 2 = 5 total mutations, 1/5 = 0.20 × 3 FP mutations = 0.6 FP and 0.20 × 2 FN mutations = 0.4 FN. Concordance rate ([(true positive + true negative)/n]), sensitivity and specificity were calculated. Plasma predictive value was calculated as the number of true positives divided by the total number of plasma positives. Continuous variables are expressed in median and interquartile range if non-normally distributed and assessed by Mann-Whitney U test between two independent samples. Analyses were performed using the SPSS Statistics version 19 (IBM Corp).

## Additional Information

**How to cite this article**: Chen, K.-Z. *et al*. Circulating Tumor DNA Detection in Early-Stage Non-Small Cell Lung Cancer Patients by Targeted Sequencing. *Sci. Rep.* **6**, 31985; doi: 10.1038/srep31985 (2016).

## Supplementary Material

Supplementary Information

## Figures and Tables

**Table 1:** Clinical features of 58 lung cancer patients.

Characteristic	Parameter value
Age, years	
Mean (SD)	64.5 (9.3)
Median (Range)	64 (40–84)
Sex, n (%)	
Male	33 (56.9)
Female	25 (43.1)
Pathological diagnosis, n (%)	
Non-small cell lung cancer	58 (100.0)
Adenocarcinoma (AC)	51 (87.9)
Squamous cell carcinoma (SCC)	7 (12.1)
Tumor Stage, n (% in all samples)	
IA	30 (52.0)
IB	16 (28.0)
IIA	12 (21.0)
Smoking status, n (% in all samples)	
Non-smoker	36 (62.1%)
Smoker	22 (37.9%)

**Table 2:** Correlations between cfDNA concentrations and clinical features.

Clinical feature	Parameter value	n	cfDNA concentration avg ± SD (ng/ml)	p-value^a^
Age	<65	27	5.64 ± 10.71	0.303
	≥65	31	7.4 ± 11.54	
Sex	Female	25	9.6 ± 13.42	0.318
	Male	33	4.29 ± 8.47	
GGO	N	50	7.53 ± 11.69	0.05
	Y	8	0.66 ± 0.7	
Differentiation level	Median or high	44	5.35 ± 9.16	0.138
	Poor	14	10.44 ± 15.53	
Vascular invasion	N	51	6.46 ± 11.17	0.383
	Y	7	7.49 ± 11.41	
VPI	N	48	6.11 ± 10.6	0.458
	Y	10	8.85 ± 13.65	
Histology	SCC	7	10.11 ± 12.93	0.671
	AC	51	5.66 ± 10.54	
Stage	I	46	4.57 ± 7.74	0.05
	II	12	14.28 ± 17.66	

GGO: ground glass opacity; VPI: visceral pleural involvement; SCC: squamous cell carcinoma; AC: adenocarcinoma.

^a^Mann-Whitney U test.
