# Supplementary material for: Circulating Tumor DNA Detection in Early-Stage Non-Small Cell Lung Cancer Patients by Targeted Sequencing
Source: Sci Rep. 2016 Aug 24;6:31985. doi: 10.1038/srep31985 (PMC4995492; doi:10.1038/srep31985)
Supplement: Supplementary Information [file srep31985-s1.doc]

**Circulating Tumor DNA Detection in Early-Stage Non-Small Cell Lung Cancer Patients by Targeted Sequencing**

Ke-Zhong Chen^#^, Feng Lou^#^, Fan Yang^#^, Jing-Bo Zhang, Hua Ye, Wei Chen, Tian Guan, Ming-Yu Zhao, Xue-Xia Su, Rong Shi, Lindsey Jones, Xue F. Huang, Si-Yi Chen^*^, and Jun Wang^*^

**
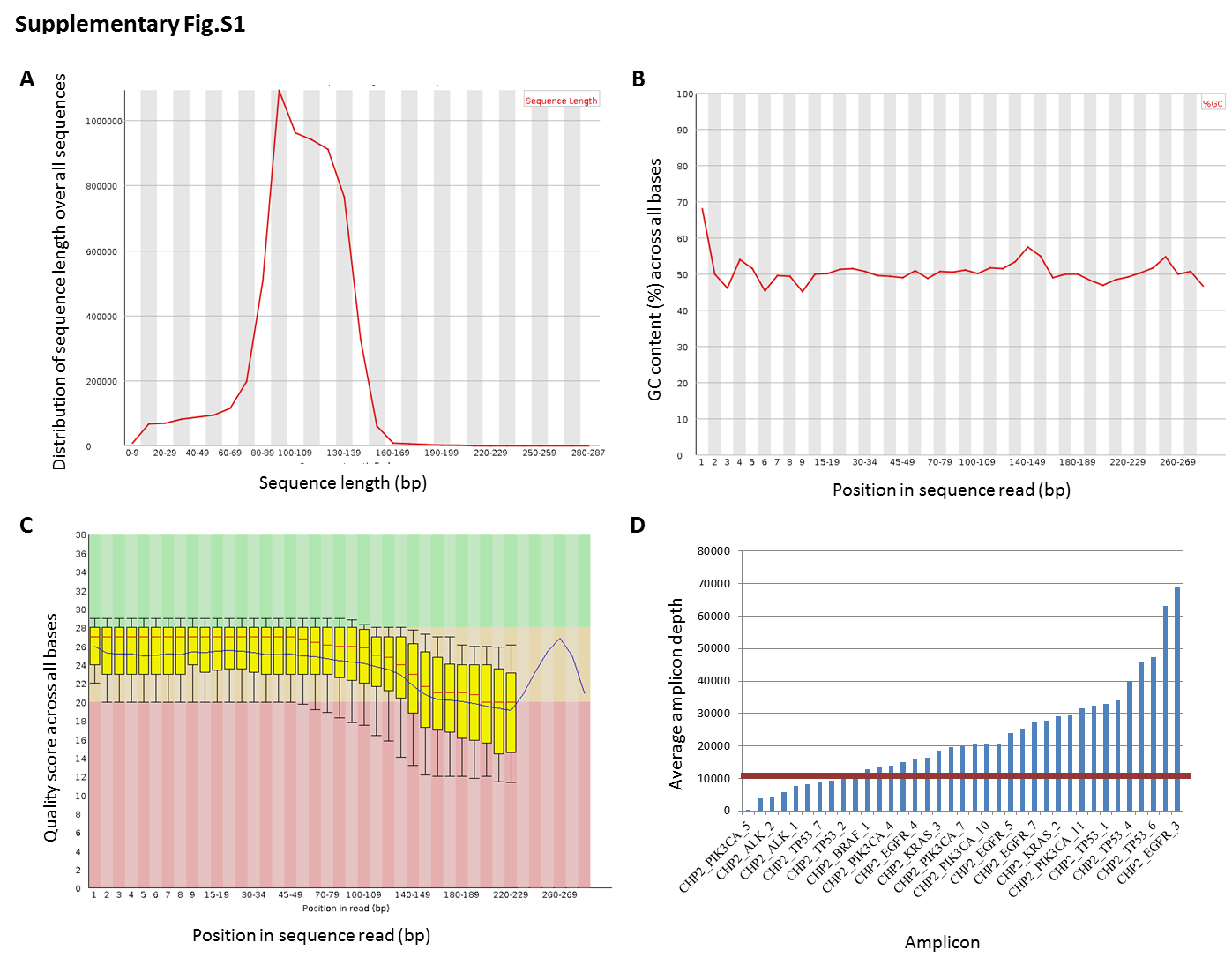
**

**Supplementary Fig.S1.** Sequencing coverage. **A**) Distribution of sequence lengths over all sequences of plasma ctDNA. The sequence length distribution largely fit the normal distribution, and most lengths are between 60 to 160 bp. Therefore, the sequences in this range of lengths were selected for further analysis; **B**) GC content across all bases of plasma ctDNA. The region of reads between 1 to 20 bp fluctuates widely and was therefore removed during quality control; **C**) Quality scores across all bases of plasma ctDNA. The quality score of every base called during the sequencing runs illustrates the sequencing accuracy; **D**) The depth of each amplicon of all plasma samples, where the depth of most amplicons is over 10,000x.

**Supplementary Table S1.** Sequencing results from four healthy individuals.

| SVID | Chromosome | Position | Gene Symbol | hotspot ID | Mutation frequency (reads) |
| --- | --- | --- | --- | --- | --- |
| SGTC_CA50_AT | chr9 | 21970987 | *CDKN2A* | COSM13608 | 100.00% (3329) |
| SGTC_CA50_AT | chr11 | 534242 | *HRAS* | COSM249860 | 46.25% (9984) |
| SGTC_CA50_AT | chr17 | 7579472 | *TP53* | COSM45985 | 41.84% (9989) |
| SGTC_CA50_AT | chr5 | 112175769 | *APC* | COSM19714 | 51.24% (9782) |
| SGTC_CA50_AT | chr5 | 112175769 | *APC* | COSM19349 | 51.24% (9782) |
| SGTC_CA50_AT | chr4 | 55141050 | *PDGFRA* | COSM12417 | 100.00% (9748) |
| SGTC_CA50_AT | chr9 | 21970987 | *CDKN2A* | COSM33797 | 100.00% (3329) |
| SGTC_CA50_BT | chr11 | 534242 | *HRAS* | COSM249860 | 98.86% (9989) |
| SGTC_CA50_BT | chr17 | 7579472 | *TP53* | COSM45985 | 95.90% (9991) |
| SGTC_CA50_BT | chr5 | 112175769 | *APC* | COSM19714 | 100.00% (9903) |
| SGTC_CA50_BT | chr5 | 112175769 | *APC* | COSM19349 | 100.00% (9903) |
| SGTC_CA50_BT | chr4 | 55141050 | *PDGFRA* | COSM12417 | 100.00% (9738) |
| SGTC_CA50_CT | chr22 | 24176287 | *SMARCB1* | COSM1090 | 50.50% (10000) |
| SGTC_CA50_CT | chr19 | 1223125 | *STK11* | COSM21360 | 50.30% (9134) |
| SGTC_CA50_CT | chr17 | 7579472 | *TP53* | COSM45985 | 44.29% (9987) |
| SGTC_CA50_CT | chr5 | 112175769 | *APC* | COSM19714 | 100.00% (9902) |
| SGTC_CA50_CT | chr5 | 112175769 | *APC* | COSM19349 | 100.00% (9902) |
| SGTC_CA50_CT | chr4 | 55141050 | *PDGFRA* | COSM12417 | 100.00% (9697) |
| SGTC_CA50_DT | chr11 | 534242 | *HRAS* | COSM249860 | 100.00% (9897) |
| SGTC_CA50_DT | chr19 | 1223125 | *STK11* | COSM21360 | 50.93% (9990) |
| SGTC_CA50_DT | chr17 | 7579472 | *TP53* | COSM45985 | 96.76% (9989) |
| SGTC_CA50_DT | chr5 | 112175769 | *APC* | COSM19714 | 40.37% (9860) |
| SGTC_CA50_DT | chr5 | 112175769 | *APC* | COSM19349 | 40.37% (9860) |
| SGTC_CA50_DT | chr4 | 55141050 | *PDGFRA* | COSM12417 | 100.00% (9692) |

**Supplementary Table S2.** Concordant mutations detected in matched tDNA and plasma ctDNA. (AC: adenocarcinoma; SCC: squamous cell carcinoma; SNP: single nucleotide polymorphism; DEL: deletion)

| **Patient no.** | **Cancer Type,**  **Stage** | **Smoking**  **(Y/N)** | **Gene** | **Mutation** | **Mut. Type** | **% Mutation: Tumor (reads)** | **% Mutation: Plasma (reads)** | **% Mutation: WBC (reads)** |
| --- | --- | --- | --- | --- | --- | --- | --- | --- |
| 16 | AC, IA | N | *SMAD4* | p.R445R | SNP | 9.64% (498) | 11.0% (21192) | 0.0% (1218) |
| 18 | AC, IA | Y | *TP53* | p.T245P | SNP | 18.94% (1811) | 0.25% (13795) | 0.0% (1196) |
| 50 | AC, IB | N | *TP53* | p.K93E | SNP | 15.59% (712) | 0.21% (4679) | 0.0% (6408) |
|  |  |  | *EGFR* | p.745_750del | DEL | 67.70% (2793) | 0.38% (21675) | 0.0% (10334) |
| 51 | SCC, IIA | Y | *PTEN* | p.R130* | SNP | 25.22% (4727) | 1.37% (9957) | 0.0% (4642) |
| 73 | AC, IA | Y | *EGFR* | p.G719A | SNP | 12.28% (9978) | 0.47% (33818) | 0.0% (24541) |
|  |  |  | *EGFR* | p.D761Y | SNP | 7.69% (9983) | 0.44% (33824) | 0.0% (24544) |
|  |  |  | *PIK3CA* | p.E545K | SNP | 13.07% (9993) | 0.21% (33818) | 0.0% (24541) |
|  |  |  | *PIK3CA* | p.E545D | SNP | 13.10%(9992) | 0.15% (33824) | 0.0% (24544) |
| 211 | AC, IB | N | *EGFR* | p.745_750del | DEL | 11.18% (9714) | 0.39% (6989) | 0.0% (511) |
| 229 | AC, IIA | Y | *TP53* | p.R175H | SNP | 49.71% (9977) | 0.10% (66979) | 0.0% (10652) |
| 231 | AC, IIA | N | *TP53* | p.E198* | SNP | 74.47% (8089) | 0.33% (8793) | 0.0% (5811) |
| 250 | AC, IA | N | *EGFR* | p.L858R | SNP | 5.88% (9981) | 0.75% (25957) | 0.0% (12601) |
| 253 | AC, IA | N | *EGFR* | p.745_749del | DEL | 5.33% (9821) | 0.32% (11063) | 0.0% (28017) |
| 262 | AC, IA | Y | *CTNNB1* | p.S45P | SNP | 17.72% (2257) | 0.14% (20538) | 0.0% (3855) |
| 281 | AC, IIA | N | *KRAS* | p.G12V | SNP | 2.15% (8378) | 0.20% (26250) | 0.0% (2792) |
| 317 | AC, IB | N | *EGFR* | p.745_750del | DEL | 18.28% (1619) | 0.37% (26285) | 0.0% (3484) |
| 324 | AC, IB | Y | *TP53* | p.C275Y | SNP | 91.30% (6380) | 0.20% (7010) | 0.0% (3536) |
|  |  |  | *TP53* | p.P33A | SNP | 3.13% (6061) | 2.70% (3979) | 0.0% (7686) |
| 326 | SCC, IIA | Y | *KRAS* | p. T58I | SNP | 6.91% (9306) | 0.10% (9667) | 0.0% (9182) |
|  |  |  | *TP53* | p. Y181H | SNP | 12.20% (8761) | 0.10% (16884) | 0.0% (8629) |
| 327 | AC, IB | N | *EGFR* | p. V774M | SNP | 25.23% (7434) | 0.10% (4274) | 0.0% (9951) |
|  |  |  | *EGFR* | p. H773L | SNP | 25.13 (7425) | 0.10% (4280) | 0.0% (9960) |
| 335 | AC, IA | N | *EGFR* | p.L858R | SNP | 6.74% (3046) | 0.60% (9464) | 0.0% (5189) |
| 336 | AC, IB | Y | *EGFR* | p.745_750del | DEL | 18.14% (1909) | 0.30% (30407) | 0.0% (5908) |
| 338 | SCC, IA | Y | *EGFR* | p.L858R | SNP | 18.43% (2571) | 0.40% (12343) | 0.0% (7232) |
| 341 | AC, IB | N | *EGFR* | p.L858R | SNP | 4.91% (4451) | 0.70% (10111) | 0.0% (1300) |
| 343 | AC, IA | N | *EGFR* | p.L858R | SNP | 10.84% (2409) | 0.10% (9667) | 0.0% (9182) |
| 344 | AC, IA | Y | *KRAS* | p.G12C | SNP | 11.18% (2319) | 0.36% (11872) | 0.0% (2559) |
| 358 | AC, IA | N | *EGFR* | p.745_750del | DEL | 15.80% (23785) | 15.78% (8206) | 0.0% (2480) |

**Supplementary Table S3.** Discordant mutations detected in tDNA only. (AC: adenocarcinoma; SCC: squamous cell carcinoma; SNP: single nucleotide polymorphism; MNP: multi-nucleotide polymorphism; DEL: deletion)

| **Patient no.** | **Cancer Type,**  **Stage** | **Smoking**  **(Y/N)** | **Gene** | **Mutation** | **Mut. Type** | **% Mutation: Tumor (reads)** | **% Mutation: Plasma (reads)** | **% Mutation: WBC (reads)** |
| --- | --- | --- | --- | --- | --- | --- | --- | --- |
| 51 | SCC, IIA | Y | *TP53* | p.V157F | SNP | 31.93% (9947) | 0.03% (28400) | 0.0% (1399) |
| 52 | AC, IA | N | *EGFR* | p.747_753del | DEL | 10.46% (4379) | 0.04% (27212) | 0.0% (13154) |
| 63 | SCC, IA | Y | *TP53* | p.H179Y | SNP | 40.01% (9983) | 0.06% (42390) | 0.0% (8504) |
|  |  |  | *KRAS* | p.G12V | SNP | 21.16% (9993) | 0.02% (31944) | 0.0% (7794) |
| 229 | AC, IIA | Y | *EGFR* | p.A750P | SNP | 23.20% (5910) | 0.01% (38350) | 0.0% (10342) |
|  |  |  | *EGFR* | p.745_750del | DEL | 53.58% (9748) | 0.05% (38378) | 0.0% (10365) |
| 231 | AC, IIA | N | *CDKN2A* | intron | SNP | 72.45% (646) | 0.06% (1824) | 0.0% (472) |
| 245 | AC, IIA | Y | *EGFR* | p.L858R | SNP | 16.13% (9970) | 0.03% (68050) | 0.0% (22588) |
| 249 | AC, IB | Y | *TP53* | p.V216L | SNP | 25.96% (6509) | 0.02% (20814) | 0.0% (10144) |
| 256 | AC, IA | N | *EGFR* | p.L858R | SNP | 18.36% (5219) | 0.03% (20503) | 0.0% (2268) |
| 259 | AC, IA | N | *EGFR* | p.746_749del | DEL | 43.43% (1453) | 0.02% (22163) | 0.0% (4801) |
|  |  |  | *TP53* | p.R306* | SNP | 17.54% (496) | 0.01% (4293) | 0.0% (1371) |
| 262 | AC, IA | Y | *EGFR* | p.745_750del | DEL | 21.84% (3842) | 0.06% (17150) | 0.0% (6813) |
| 264 | AC, IA | N | *EGFR* | p.745_750del | DEL | 44.04% (7917) | 0.06% (26176) | 0.0% (8142) |
|  |  |  | *TP53* | p.R234H | SNP | 22.00% (2091) | 0.05% (19904) | 0.0% (3827) |
| 278 | AC, IA | Y | *TP53* | p.R234H | SNP | 6.54% (1956) | 0.01% (8285) | 0.0% (4745) |
| 316 | AC, IA | N | *EGFR* | p.L861Q | SNP | 6.78% (5646) | 0.00% (11830) | 0.0% (12869) |
| 322 | AC, IB | N | *EGFR* | p.G719A | SNP | 5.84% (3560) | 0.08% (57607) | 0.0% (7350) |
| 326 | SCC, IIA | Y | *FBXW7* | p.R347P | SNP | 9.67% (9018) | 0.00% (27983) | 0.0% (14508) |
| 328 | SCC, IB | Y | *TP53* | p.G227X | SNP | 66.44% (1858) | 0.00% (8609) | 0.0% (9921) |
| 340 | AC, IB | N | *EGFR* | p.L858R | SNP | 27.61% (2583) | 0.03% (20516) | 0.0% (6262) |
| 343 | AC, IA | N | *KRAS* | p.GG12_13AT | MNP | 22.99% (2275) | 0.01% (12277) | 0.0% (7596) |
| 355 | AC, IIA | N | *TP53* | p.R234H | SNP | 7.60% (1763) | 0.00% (4009) | 0.0% (1060) |
|  |  |  | *PIK3CA* | p.H1047R | SNP | 4.38% (2203) | 0.03% (9616) | 0.0% (1498) |
| 360 | AC, IA | N | *EGFR* | p.L858R | SNP | 28.37% (5862) | 0.01% (16752) | 0.0% (7947) |

| **Patient no.** | **Cancer Type,**  **Stage** | **Smoking**  **(Y/N)** | **Gene** | **Mutation** | **Mutation Type** | **% Mutation: Tumor (reads)** | **% Mutation: Plasma (reads)** | **% Mutation: WBC (reads)** |
| --- | --- | --- | --- | --- | --- | --- | --- | --- |
| 16 | AC, IA | N | *TP53* | p.S144L | SNP | 0.00% (1110) | 1.86% (9994) | 0.0% (1218) |
| 18 | AC, IA | Y | *TP53* | p.S144L | SNP | 0.00% (4311) | 1.58% (9994) | 0.0% (3520) |
| 37 | AC, IB | Y | *KRAS* | p.G12V | MNP | 0.00% (517) | 2.72% (9973) | 0.0% (429) |
| 50 | AC, IB | N | *PIK3CA* | p.R108H | SNP | 0.00% (267) | 1.88% (5430) | 0.0% (10003) |
| 51 | SCC, IIA | Y | *KIT* | p.M541L | SNP | 0.01% (15649) | 1.36% (9969) | 0.0% (3046) |
| 63 | SCC, IA | Y | *TP53* | p.F231V | SNP | 0.00% (178) | 12.27% (19162) | 0.0% (3783) |
| 228 | AC, IIA | Y | *EGFR* | p.T790M | SNP | 0.00% (2532) | 1.61% (6696) | 0.0% (128) |
|  |  |  | *KRAS* | p.G12V | MNP | 0.03% (13640) | 2.85% (9884) | 0.0% (11189) |
| 246 | AC, IB | N | *EGFR* | p.747_753del | DEL | 0.00% (10721) | 4.69% (6398) | 0.0% (17230) |
|  |  |  | *EGFR* | p.L858R | SNP | 0.13% (19181) | 2.81% (9952) | 0.0% (7616) |
| 256 | AC, IA | N | *EGFR* | p.L703P | SNP | 0.17% (1731) | 1.00% (9978) | 0.0% (1285) |
|  |  |  | *TP53* | p.C143R | SNP | 0.00% (2813) | 0.99% (9997) | 0.0% (3103) |
|  |  |  | *TP53* | p.D189G | SNP | 0.34% (2634) | 1.18% (10000) | 0.0% (2711) |
|  |  |  | *TP53* | p.H140R | SNP | 0.00% (2816) | 1.03% (9993) | 0.0% (3087) |
| 259 | AC, IA | N | *PIK3CA* | p.K111R | SNP | 0.00% (307) | 1.08% (5535) | 0.0% (754) |
|  |  |  | *TP53* | p.M130V | SNP | 0.00% (1214) | 1.23% (9990) | 0.0% (5651) |
|  |  |  | *VHL* | p.R167W | SNP | 0.00% (895) | 1.07% (9958) | 0.0% (4388) |
| 263 | AC, IA | N | *PIK3CA* | p.R108H | SNP | 0.00% (644) | 1.16% (4906) | 0.0% (164) |
| 315 | AC, IA | N | *ALK* | p.R1275Q | SNP | 0.00% (249) | 4.97% (1449) | 0.0% (552) |
| 313 | AC, IA | N | *GNAS* | p.R201S | SNP | 0.00% (1526) | 1.08% (5347) | 0.0% (1206) |
| 326 | SCC, IIA | Y | *EGFR* | p.745_749del | DEL | 0.00% (15166) | 3.62% (9267) | 0.0% (12752) |
|  |  |  | *EGFR* | p.G719A | SNP | 0.00% (21175) | 1.80% (9807) | 0.0% (11632) |
|  |  |  | *EGFR* | p.D761Y | SNP | 0.00% (14837) | 3.68% (9617) | 0.0% (12618) |
|  |  |  | *EGFR* | p.P772fs | INS | - | 3.76% (7816) | 0.0% (-) |
|  |  |  | *EGFR* | p.D770fs | INS | - | 3.88% (7935) | 0.0% (-) |
|  |  |  | *EGFR* | p.S768I | SNP | 0.30% (6834) | 3.87% (7977) | 0.0% (4592) |
|  |  |  | *PIK3CA* | p.H1047R | SNP | 0.20% (11672) | 6.47% (9349) | 0.0% (8654) |
|  |  |  | *RB1* | p.R455X | SNP | 0.00% (10328) | 1.07% (9884) | 0.0% (9472) |
|  |  |  | *TP53* | p.P33R | SNP | 0.00% (14558) | 8.55% (9132) | 0.0% (11039) |
|  |  |  | *TP53* | p.A122A | SNP | 0.00% (22106) | 1.53% (9826) | 0.0% (17744) |
|  |  |  | *TP53* | p.G227X | SNP | 0.00% (5907) | 1.25% (9872) | 0.0% (7298) |
|  |  |  | *TP53* | p.G160G | SNP | 0.00% (12093) | 1.02% (9851) | 0.0% (8854) |
|  |  |  | *TP53* | p.R157X | SNP | 0.20% (12978) | 2.01% (9735) | 0.0% (8885) |
|  |  |  | *VHL* | p.L85P | SNP | 0.10% (10516) | 1.41% (9857) | 0.0% (4824) |
| 327 | AC, IB | N | *TP53* | p.P33A | SNP | 2.80% (18473) | 4.83% (7711) | 0.0% (19336) |
| 328 | SCC, IB | Y | *EGFR* | p.T790M | SNP | 0.10% (49429) | 2.33% (7263) | 0.0% (9573) |
|  |  |  | *EGFR* | p.S768I | SNP | 0.03% (49425) | 2.43% (7224) | 0.0% (9647) |
|  |  |  | *PIK3CA* | p.H1047R | SNP | 0.60% (97959) | 6.98% (9295) | 0.0% (12799) |
| 329 | AC, IA | N | *MPL* | p.A519T | SNP | 0.10% (31624) | 0.96% (9897) | 0.0% (22133) |
| 333 | AC, IIA | N | *EGFR* | p.D770fs | INS | 0.01% (8997) | 1.02% (5511) | 0.0% (-) |
|  |  |  | *PIK3CA* | p.H1047R | SNP | 1.00% (13858) | 2.13% (9785) | 0.0% (11198) |
| 336 | AC, IB | Y | *TP53* | p.A120P | SNP | 0.00% (5666) | 0.97% (9890) | 0.0% (9115) |

**Supplementary Table S4.** Discordant mutations detected in plasma ctDNA only. (AC: adenocarcinoma; SCC: squamous cell carcinoma; SNP: single nucleotide polymorphism; MNP: multi-nucleotide polymorphism; DEL: deletion; INS: insert)

**Supplementary Table S5.** Tumor biomarker levels versus cfDNA levels. The positive cut-off values for these serum biomarkers are CA125 (>35 U/ml), CA19-9 (>39 U/ml), CEA (>4.7 ng/ml), CYFRA21-1 (>3.3 ng/ml), and NSE (>16.3 ng/ml).

| **Patient no.** | **Tumor Biomarkers** | | | | | **cfDNA mutation frequency (%)** | **cfDNA concentration (ng/ml)** |
| --- | --- | --- | --- | --- | --- | --- | --- |
|  | **CA125 (U/ml)** | **CA19-9 (U/ml)** | **CEA (ng/ml)** | **CYFRA21-1 (ng/ml)** | **NSE (ng/ml)** |  |  |
| 16 | - (14.5) | - (19.68) | + (8.38) | - (2.67) | - (13.77) | 0.11 | 22.0000 |
| 18 | ND | - (<0.60) | - (3.03) | ND | ND | 0.1119 | 21.7534 |
| 37 | ND | ND | ND | ND | ND | 0.0272 | 1.7299 |
| 50 | - (12.47) | - (9.49) | - (2.02) | - (3.19) | - (9.72) | 0.0188 | 1.9101 |
| 51 | - (9.15) | - (3.55) | - (1.24) | + (6.94) | - (11.88) | 0.0137 | 0.8878 |
| 52 | - (8.86) | - (25.29) | + (5.37) | + (3.47) | - (8.65) | 0.0004 | 0.3936 |
| 63 | - (3.19) | - (9.24) | + (4.97) | + (4.58) | - (8.77) | 0.1227 | 21.3989 |
| 73 | - (12.27) | - (11.48) | - (2.55) | + (4.42) | - (10.9) | 0.0047 | 2.4440 |
| 191 | - (16.63) | - (17.27) | - (2.77) | - (1.13) | + (26.45) | 0 | 39.5760 |
| 194 | - (7.83) | - (12.48) | - (2.84) | - (1.48) | - (9.84) | 0 | 0 |
| 211 | - (8.22) | - (7.73) | - (3.44) | - (2.51) | - (11.11) | 0.0039 | 1.7004 |
| 216 | ND | ND | ND | ND | ND | 0 | 26.6800 |
| 228 | - (7.39) | - (9.74) | - (2.98) | - (1.7) | - (6.47) | 0.0285 | 3.6024 |
| 229 | - (15.79) | - (38.53) | - (3.81) | + (7.09) | - (11.07) | 0.001 | 0.1040 |
| 231 | - (13.1) | - (1.28) | - (3.47) | + (10.95) | - (13.82) | 0.0033 | 0.9187 |
| 245 | - (19.1) | + (41.19) | + (5.35) | - (1.42) | - (13.27) | 0.0003 | 0.0636 |
| 246 | - (19.63) | - (15.42) | - (2.24) | - (1.51) | - (12.9) | 0.0469 | 8.3669 |
| 249 | - (15.35) | - (12.3) | - (2.49) | - (1.75) | - (13.99) | 0.0019 | 0.3602 |
| 250 | ND | - (10.48) | + (15.61) | - (1.88) | - (9.64) | 0.0075 | 0.7380 |
| 253 | - (8.52) | - (12.17) | - (1.15) | - (2.08) | - (15.42) | 0.0032 | 0.5811 |
| 256 | - (6.59) | - (18.47) | + (6.4) | - (1.05) | - (13.72) | 0.207 | 31.9608 |
| 259 | - (15.62) | - (7.08) | - (3.05) | - (1.85) | - (11.19) | 0.0123 | 2.8733 |
| 261 | - (9.98) | - (6.19) | - (2.11) | + (5.89) | - (15.57) | 0 | 38.7092 |
| 262 | - (7.89) | - (16.38) | - (2.1) | - (1.68) | - (11.53) | 0.0014 | 0.3774 |
| 263 | - (21.97) | - (23.65) | - (2.81) | - (2.26) | + (16.94) | 0.0116 | 0.5429 |
| 264 | - (6.45) | - (13.33) | - (3.27) | + (3.65) | - (11.85) | 0.0006 | 0.0710 |
| 278 | ND | ND | - (2.06) | + (4.28) | - (8.66) | 0.0001 | 0.0245 |
| 279 | - (3.93) | - (3.74) | - (2.21) | + (3.65) | - (9.96) | 0 | 0.3433 |
| 281 | ND | - (12.35) | - (1.63) | + (3.48) | - (9.74) | 0.002 | 0.6272 |
| 282 | - (7.98) | - (10.59) | - (1.36) | - (3.01) | - (13.12) | 0 | 0.3739 |
| 298 | - (8.49) | - (8.61) | - (0.8) | - (3.15) | - (12.74) | 0 | 10.1868 |
| 311 | - (10.46) | ND | ND | - (1.98) | - (12.13) | 0 | 0 |
| 310 | - (7.49) | - (3.77) | - (1.47) | - (2.06) | - (13.71) | 0 | 0 |
| 313 | - (13.14) | - (10.33) | - (1.49) | - (1.42) | - (12.04) | 0.0108 | 2.3501 |
| 314 | ND | - (12.08) | - (2.42) | + (3.36) | - (12.3) | 0 | 0 |
| 315 | - (10.48) | - (8.25) | - (1.32) | + (4.1) | - (9.59) | 0.0497 | 14.1943 |
| 316 | - (11.15) | - (6.18) | - (1.85) | - (2.15) | - (11.02) | 0 | 0 |
| 317 | - (12.78) | - (11.93) | - (4.37) | - (1.28) | - (11.61) | 0.0037 | 0.6349 |
| 322 | - (8.22) | - (7.73) | - (3.44) | - (2.51) | - (11.11) | 0.0008 | 0.2707 |
| 323 | ND | ND | ND | ND | ND | 0 | 0.3868 |
| 324 | - (13.04) | - (11.96) | - (2.48) | - (3.12) | + (16.79) | 0.027 | 5.8320 |
| 326 | ND | - (26.29) | - (4.69) | + (4.24) | - (11.28) | 0.0855 | 10.4652 |
| 327 | - (7.5) | - (11.56) | - (2.17) | - (3.19) | - (12.16) | 0.0483 | 12.7512 |
| 328 | - (7.32) | - (12.15) | - (2.04) | - (1.83) | - (9.08) | 0.0698 | 15.8586 |
| 329 | - (8.13) | - (20.4) | - (4.57) | - (1.45) | - (10.46) | 0.0096 | 1.1443 |
| 333 | - (10.54) | - (26.21) | + (12.43) | + (3.49) | - (9.3) | 0.0213 | 5.6062 |
| 335 | - (7.29) | - (12.98) | - (1.26) | - (1.36) | - (13.48) | 0.006 | 1.5168 |
| 336 | - (13.3) | - (14.33) | - (1.04) | - (1.54) | - (13.7) | 0.0097 | 1.6994 |
| 338 | - (5.46) | - (8.75) | - (3.36) | - (0.95) | - (11.88) | 0.004 | 1.0592 |
| 339 | ND | - (15.93) | + (4.98) | - (3.21) | - (11.24) | 0 | 0.3934 |
| 340 | - (10.39) | - (12.82) | - (2.13) | - (2.13) | - (9.28) | 0.0003 | 0.0864 |
| 341 | - (11.59) | - (17.83) | - (4.08) | - (2.36) | - (12.08) | 0.007 | 2.1504 |
| 343 | ND | - (19.95) | - (1.98) | - (1.17) | - (10.1) | 0.001 | 0.3320 |
| 344 | - (7.26) | - (9.07) | - (2.78) | - (2.02) | - (14.8) | 0.0036 | 0.7373 |
| 355 | - (11.12) | + (112.4) | - (3.43) | + (5.33) | - (13.28) | 0.3805 | 44.1096 |
| 356 | - (10.24) | ND | - (3.52) | ND | ND | 0 | 0 |
| 358 | - (15.25) | - (19.27) | - (1.38) | - (0.81) | + (16.82) | 0.1578 | 18.7456 |
| 360 | - (15.49) | - (<0.60) | - (4.66) | - (2.02) | - (12.53) | 0.0001 | 0.02216 |
